# Patient stratification and genomics: flares, fizzlers and foxes

**DOI:** 10.1186/1479-5876-10-S3-I5

**Published:** 2012-11-28

**Authors:** Kenneth GC Smith

**Affiliations:** 1 Cambridge Institute for Medical Research and Dept. of Medicine, University of Cambridge School of Clinical Medicine, Addenbrooke’s Hospital, Cambridge, UK

## 

Diseases have traditionally been defined predominantly by clinical criteria, sometimes with the assistance of “conventional” laboratory data. Recent advances in genomic technology have begun to find previously undiscovered sub-divisions within what were thought to be single diagnoses, sometimes with significant clinical implications. This talk will discuss the potential for such technologies to redefine the classification and treatment of autoimmune diseases, using two recent examples from Cambridge.

The first is the use of a Genome Wide Association Study to determine the genetic underpinning of ANCA-associated Vasculitis (AAV). This study has confirmed a genetic component to AAV pathogenesis, and demonstrated that the AAV clinical syndromes granulomatosis with polyangiitis (GPA: formerly Wegener’s) and microscopic polyangiitis (MPA) are genetically distinct diseases better defined by ANCA specificity for proteinase-3 (PR3) or myeloperoxidase (MPO) rather than clinical syndrome. In addition the response against the autoantigen PR3 (encoded by *PRTN3*) was found to be a central etiological feature of PR3-ANCA associated vasculitis. Thus PR3- and MPO-ANCA-associated vasculitis must be considered as distinct autoimmune syndromes [1].

The second example will describe the use of whole genome transcriptome analysis of purified CD8 T cells to define two sub-groups within patients with a number of inflammatory diseases (including SLE, AAV, Crohn’s disease and ulcerative colitis). These otherwise “invisible” groups have markedly different long term outcomes [2,3]. This has implications for therapy, and has led to the development of a biomarker now entering clinical trials. Follow-up studies have led to the discovery of a new inflammatory pathway controlling TNFα and IL-10 production that might drive this difference in outcome.
